# Trend and risk factors of low birth weight and macrosomia in south China, 2005–2017: a retrospective observational study

**DOI:** 10.1038/s41598-018-21771-6

**Published:** 2018-02-21

**Authors:** Jiaming Rao, Dazhi Fan, Shuzhen Wu, Dongxin Lin, Huishan Zhang, Shaoxin Ye, Xin Luo, Lijuan Wang, Jianwei Yang, Minhui Pang, Jiayi Zhang, Qing Xia, Xiaoke Yang, Wen Wang, Yao Fu, Yan Liu, Xiaoling Guo, Zhengping Liu

**Affiliations:** 1 Foshan Institute of Fetal Medicine, Southern Medical University Affiliated Maternal & Child Health Hospital of Foshan, Foshan, Guangdong, 528000 China; 2 Department of Obstetrics, Southern Medical University Affiliated Maternal & Child Health Hospital of Foshan, Foshan, Guangdong 528000 China; 3 Department of Epidemiology, School of Basic Medicine, Jinan University, Guangzhou, Guangdong, 510632 China; 4 Department of Epidemiology and Biostatistics, School of Public Health, Anhui Medical University, Hefei, Anhui 230032 China; 5 Menzies Institute for Medical Research, University of Tasmania, Private Bag 23, Hobart, Tasmania 7000 Australia; 6 Department of Rheumatism and Immunity, the First Affiliated Hospital of Anhui Medical University, Hefei, Anhui 230000 China

## Abstract

The percentages of low birth weight (LBW) increased from 7.7% in 2005 to 11.3% in 2011 and declined to 8.1% in 2017. For very low birth weight (VLBW) individuals, the proportion declined −1.0% annually, from 2.5% in 2005 to 1.4% in 2017. Among moderately low birth weight (MLBW) individuals, the proportion first increased 12.8% annually, from 5.0% in 2005 to 9.3% in 2011, and then declined −3.8% annually, from 9.4% in 2011 to 7.0% in 2017. The percentages of macrosomia monotone decreased from 4.0% in 2005 to 2.5% in 2017, an annual decline of −4.0%. Multiple regression analyses showed that boys, maternal age, hypertensive disorders complicating pregnancy (HDCP), and diabetes were significant risk factors for LBW. Boys, maternal age, gestational age, HDCP, diabetes, and maternal BMI were significant risk factors for macrosomia. Although the relevant figures declined slightly in our study, it is likely that LBW and macrosomia will remain a major public health issue over the next few years in China. More research aimed at control and prevention of these risk factors for LBW and macrosomia and their detrimental outcome in the mother and perinatal child should be performed in China.

## Introduction

Low birth weight (LBW, <2500 g) is a most important factor of neonatal mortality and a major determinant of postnatal infant mortality, as well as morbidity in infancy and in older childhood^1^. Studies have shown that LBW infants die 40 times as much as normal weight infants, and LBW infants are many times more likely to end up with long-term handicapping conditions^2^. Children with LBW increased over the past two decades in many countries^3–6^. The prevalence of LBW is estimated to be 19% in developing countries and 5% to 7% in developed countries. According to Report of National Health Services Survey of China, the proportion of low birth weight has a ratio of 2.7% in 2006, and continuously increased with the scale-up of cities. Another research^7^ including 39 hospital in 14 different provinces in China reported the incidence of LBW in mainland China was 6.1%. The risk factors for LBW, such as older maternal age, anemia, hypertension, and poor nutritional status, are widely reported in recent studies^2,3,8,9^.

Macrosomia refers to the body weight reaching or exceeding 4000 g, which can increase the risks of a poor pregnancy outcome in mothers and newborns^10,11^. Macrosomia are associated with increased risk of cesarean delivery, abnormal hemorrhage, shoulder dystocia, and post-term pregnancy, as well as keep the babies at a risk of perinatal asphyxia, fracture, cerebral hemorrhage even death^12,13^. Besides, studies have shown that there is a higher risk of developing obesity and metabolic disorders of macrosomia infants^14^. The proportion of macrosomia increased over the past thirty years in many countries^15–18^, with frequency ranging from 0.5% to 14.9% in Asia, Africa and Latin America^10^. In particular, the number in China noted an increased from 6.0% in 1994 to 7.8% in 2005^15^. There is an increase of 15–25% reported in the past three decades mainly due to an increase of maternal obesity and diabetes^19^. It is strong evidence to show that male, older maternal age, maternal diabetes, gestational body-mass index (BMI) and higher parity were significantly associated with increased odds for macrosomia in all regions^10,20^.

There are many studies focus on the incidence and risk factors of low birth weight and macrosomia, but little data on their long-term trends and their continuous observations in China. Besides, the etiology of low birth weight and macrosomia cannot be thoroughly explained by current researchs, so more observational studies are necessary. The purpose of our study was to examine the secular trend of LBW and macrosomia in Foshan, a city located in the south China, and explored relevant factors associated with LBW and macrosomia, to provide valuable information to reduce maternal and neonatal morbidity and mortality due to LBW and macrosomia.

## Results

### Characteristics of study population

The entire study population consisted of 102,526 individuals with a mean age ± SD of 28.3 ± 4.8 y. 25 to 29 year olds had the highest proportion of all individuals, reaching 43.2%, followed by 30 to 34 years (24.1%) and 20 to 24 years (20.0%). 61.7% of the study population were primiparae. The vast majority (98.6%) were Han Chinese. Clinical characteristics of the study population are described in Table 1.

**Table 1:** Characteristics of patients between 2005 and 2017.

Characteristic	N (%)	Characteristic	N (%)
Live births, n, (%)	102526(100.0)	Gestational age distribution, n (%)	
Sex		<37 wk	9294(10.6)
Boys	54759(52.5)	Apgar score at 5 minutes	
Mean age ± SD, years	28.3 ± 4.8	<7	2896(2.7)
Age, n (%)		7–10	104363(97.3)
<20 y	1459(1.4)	BMI	
20–24 y	21483(20.0)	<25	44087(44.3)
25–29 y	46316(43.2)	25 to 30	46376(46.6)
30–34 y	25869(24.1)	≥30	9056(9.1)
>35 y	12113(11.3)	HDCP	
Nationality		No	99215(96.3)
Han Chinese	101,090(98.6)	Yes	3812(3.7)
Other	1435(1.4)	Diabetes	
Parity		No	91890(90.0)
0	64456(61.7)	Yes	10210(10.0)
1	34684(33.2)	Mode of delivery	
2	4762(4.6)	Vaginal	54625(61.7)
≥3	594(0.6)	Cesarean section	45329(45.3)

### Trends in incidence of weight subgroups

**Figure 1:**
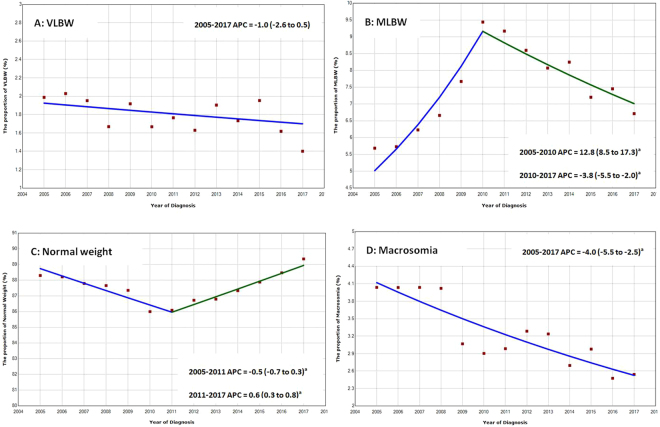
Annual Percent Change (APC) by birth weight subgroups in Foshan during 2005 to 2017. Lines were fitted according to the Joinpoint Regression Program (National Cancer Institute), version 4.4.0.0, which uses permutation analysis to fit a series of straight lines on a logarithmic scale to observed rates, indicated by circles; up to 3 joinpoints (4 line segments) were allowed. ^a^Indicates that the Annual Percent Change (APC) is significant different from zero at the alpha = 0.05 level.

Among VLBW individuals, the proportion of which declined by −1.0% annually (95% CI, −2.6% to 0.5%), from 2.5% in 2005 to 1.4% in 2017. Among MLBW individuals, the proportion of which increased by 12.8% annually (95% CI, 8.5% to 17.3%), from 5.0% in 2005 to 9.3% in 2011, and declined by −3.8% annually (95% CI, −5.5% to −2.0%), from 9.4% in 2011 to 7.0% in 2017. The proportion of normal neonatal weight remained relatively stable, ranged 85.0% to 89.3%, then declined by −0.5% annually (95% CI: −0.7 to −0.3) during 2005 to 2011 and increased by 0.6% (95% CI: 0.3 to 0.8) annually during 2011 to 2017. The proportion of macrosomia has declined from 4.1% to 2.5%, among which declined by −4.0% annually (95% CI: −5.5 to 2.5) during this period (Fig. 1).

### Trends in average weight of weight subgroups

The proportion of VLBW, MLBW, normal and macrosomia was 1.8%, 7.6%, 87.5% and 3.1% in the total population, respectively. The mean neonatal weight for VLBW, MLBW, normal and macrosomia was 1184 (SD:201), 2142 (SD:267), 3182 (SD:342) and 4198 (SD:447) grams, respectively (Table 2).

**Table 2:** The proportion and mean neonatal weight of weight subgroups.

	All	2005 to 2011	2012 to 2017
The proportion of weight subgroups (%)			
LBW	8949(8.7)	2723(8.6)	5226(8.8)
VLBW	1693(1.8)	795(1.8)	898(1.8)
MLBW	7145(7.6)	3291(7.6)	3854(7.6)
Normal	82323(87.5)	37967(87.1)	44356(87.8)
Macrosomia	2933(3.1)	1516(3.5)	1417(2.8)
Neonatal weight age ± SD, grams			
VLBW	1184 ± 201	1177 ± 200	1212 ± 316
MLBW	2142 ± 267	2116 ± 263	2166 ± 271
Normal	3182 ± 342	3166 ± 345	3194 ± 340
Macrosomia	4198 ± 447	4194 ± 336	4184 ± 194

**Figure 2:**
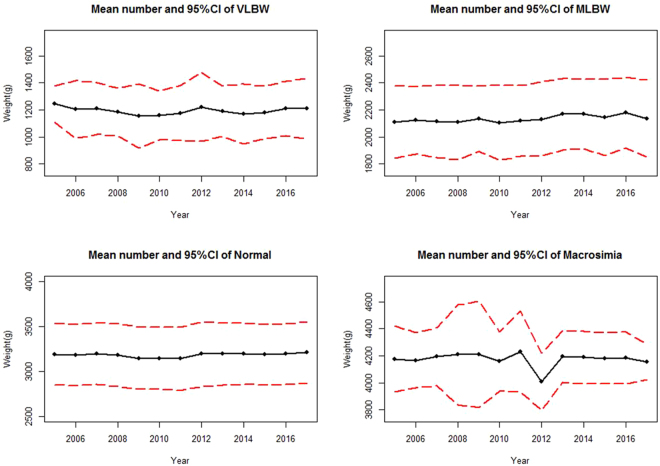
Mean number and 95% CI by birth weight subgroups in Foshan during 2005 to 2017.

The mean weight of VLBW group in the whole population between 2005 and 2017 was 1243 grams (95% CI 1108 to 1377) and 1209 grams (95% CI 984 to 1434).The mean weight of MLBW group between 2005 to 2017 has slightly increased from 2110 grams (95% CI 1842 to 2378) to 2136 grams (95% CI 1853 to 2419). The mean weight of Normal group between 2005 and 2017 was 3192 grams (95% CI 2848 to 3536) and 3210 grams (95% CI 2870 to 3549). The mean weight of macrosomia group between 2005 and 2017 was 4176 grams (95% CI 3933 to 4419) and 4353 grams (95% CI 4021 to 4284) (Fig. 2).

**Figure 3:**
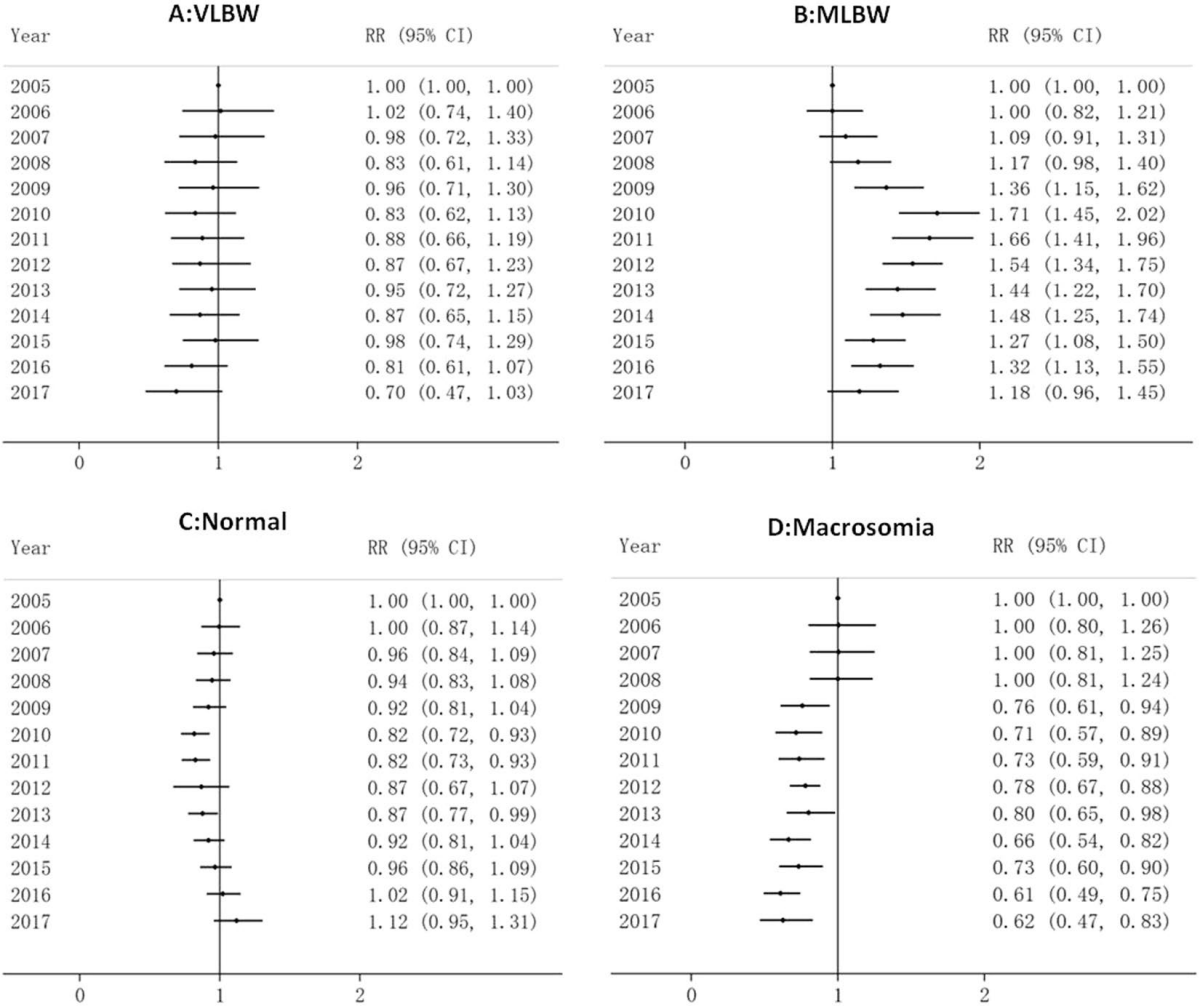
Relative risk for birth weight subgroup in Foshan during 2005 to 2017. 2005 is the reference year (RR = 1.0), adjusted for maternal age, parity and gestational age, mode of delivery, HDCP, diabetes, and maternal BMI.

From 2005 to 2017, there was a significant decrease in VLBW and macrosomia, with RR of 0.70 (95% CI: 0.47 to 1.03) and 0.62 (95% CI: 0.47 to 0.83) in 2017 respectively, reference to 2005 (RR = 1), after adjusted for maternal age, parity and gestational age. The relative risk of MLBW rose rapidly at first during 2005 to 2010, with a peaked in 2010 (RR = 1.71, 95% CI: 1.45 to 2.02), and then declined slowly during 2010 to 2017 (RR = 1.18, 95% CI: 0.96 to 1.45). The RR value of normal weight infants dropped first and then increased, and the RR values were slightly increased in 2017 (RR = 1.12, 95% CI: 0.95 to 1.31), comparing with 2005 (Fig. 3).

### Multiple regression analysis

Boys (OR = 1.17, 95% CI: 1.08–1.26, *p* < 0.001), maternal age 20 to 24 years (OR = 1.19, 95% CI: 1.57–2.67, *p* < 0.001), maternal age 25 to 29 years (OR = 2.78, 95% CI: 2.14–3.61, *p* < 0.001), maternal age 30 to 34 years (OR = 2.63, 95% CI: 2.01–3.45, *p* < 0.001), maternal age >35 years (OR = 1.85, 95% CI: 1.35–2.46, *p* < 0.001), HDCP (OR = 5.92, 95% CI: 5.35–6.55, *p* < 0.001), diabetes (OR = 1.38, 95% CI: 1.26 to 1.50, *p* < 0.001) were significant risk factors for LBW in the multiple logistic regression model.

Boys (OR = 1.91, 95% CI: 1.71–2.14, *p* < 0.001), maternal age 30 to 34 years (OR = 2.29, 95% CI: 1.17–4.47, *p* < 0.001), maternal age >35 years (OR = 2.44, 95% CI: 1.23–4.82, *p* < 0.001), HDCP (OR = 1.31, 95% CI: 1.02–1.68, *p* < 0.001), diabetes (OR = 1.58, 95% CI: 1.36 to 1.83, *p* < 0.001), gestational age during 37 to 41 weeks (OR = 6.64, 95% CI: 5.21 to 8.44, *p* < 0.001), gestational age ≥42 weeks (OR = 20.45, 95% CI: 7.63 to 54.78, *p* < 0.001), BMI 25 to 30 (OR = 3.22, 95% CI: 2.69 to 3.84, *p* < 0.001), BMI > 30 (OR = 9.62, 95% CI: 7.91 to 11.72, *p* < 0.001) were significant risk factors for macrosomia in the multiple logistic regression model (Table 3).

**Table 3:** Multiple logistic regression analyses of factors associated with LBW and Macrosomia. Adjusted for nationality, mode of delivery.

Risk Fators	LBW		Macrosomia	
	RR (95% CI)	*p*-Value	RR (95% CI)	*p*-Value
Sex				
Girls (Ref)	1		1	
Boys	1.17(1.08 to 1.26)	<0.001	1.91(1.71 to 2.14)	<0.001
Maternal age, n (%)				
<20 y (Ref)	1		1	
20–24 y	2.05(1.57 to 2.67)	<0.001	1.19(0.60 to 2.32)	
25–29 y	2.78(2.14 to 3.61)	<0.001	1.71(0.88 to 3.33)	
30–34 y	2.63(2.01 to 3.45)	<0.001	2.29(1.17 to 4.47)	<0.001
>35 y	1.85(1.39 to 2.46)	<0.001	2.44(1.23 to 4.82)	<0.001
Parity				
0 (Ref)	1		1	
1	0.92(0.84 to 1.01)	0.093	1.77(1.59 to 1.98)	<0.001
≥2	0.70(0.57 to 0.85)	<0.001	2.28(1.74 to 2.98)	<0.001
Gestational age				
<37 wk (Ref)	1		1	
37 to 41 wk	0.04(0.03 to 0.04)	<0.001	6.64(5.21 to 8.44)	<0.001
≥42 wk	0.04(0.01 to 0.38)	0.004	20.45(7.63 to 54.78)	<0.001
HDCP				
No (Ref)	1		1	
yes	5.92(5.35 to 6.55)	<0.001	1.31 (1.02 to 1.68)	0.034
Diabetes				
No (Ref)	1		1	
Yes	1.38 (1.26 to 1.50)	<0.001	1.58 (1.36 to 1.83)	<0.001
BMI				
<25 (Ref)	1		1	
25 to 30	0.56(0.51 to 0.62)	<0.001	3.22(2.69 to 3.84)	<0.001
≥30	0.68(0.58 to 0.82)	<0.001	9.62(7.91 to 11.72)	<0.001

## Discussion

Many studies have focused on the incidence and risk factors of LBW and macrosomia, but there is little data on the long-term trends and ongoing observations in China. In this study, we determined the proportion of LBW and macrosomia in Foshan during 2005 to 2017, and identified the independent risk factors of LBW and macrosomia using a logistic regression model. The proportion of VLBW monotone decreased from the years 2005 to 2017, at an annual 1.0% of decline. However, the proportion of MLBW first increased and then declined during the same period, which resulted in a slight increase in the incidence of total LBW from 7.7% to 8.1% between 2005 and 2017. A national survey showed infants with birth weights less than 1500 g (VLBW) accounted for 3.8% in 1998, in the major cities of China^21^, which was almost twice that of our study (1.8%), and the incidence of LBW infants has been between 3.3% and 4.9% in Beijing urban area in the past 20 years^5^, which was slightly lower than reported in our study. On one hand, this reduction reflects dramatic advances in medical technology and continuously improved expectant management for LBW births over the past 20 years in China. On the other hand, differences in case definition between studies, regions and nations, and the institution based tertiary care settings of many previous studies may also have played a role in the observed reduced incidence. According to World Health Organization public data^22^, the incidence rates of LBW infants in major western countries respectively were as follows: Australia (7%), Canada (6%), Germany (7%), France (7%) and US (8%) respectively, which were similar or slightly lower to our results. Furthermore, our data showed that more than 70.1% of the low birth weight infants were preterm births, which were similar to previous studies in Beijing^5^. We consider the main reason for this pattern was due to the increasing survival rates of preterm births and the extremely LBW newborns in China; therefore, it is reasonable to assume that preterm birth was the primary cause of LBW in our study. In addition, this hospital is an emergency care center for the emergency care of critically ill mothers and newborns in the city. Full term LBW infants should also deserve public attention, because these babies or their mothers maybe more likely to develop severe complications or have a worse outcome.

Epidemiological studies show that a 1000 g reduction in birth weight results in a 1.7 fold increase in the risk of proteinuria in children, and a 3-fold increased risk of proteinuria in adulthood^23^. Other studies^2,7^ have shown that LBW infants have an increased risk of hypertension, kidney disease, type 2 diabetes, obesity, and coronary heart disease; however, the etiology for these complications remains unclear. LBW infants include infants who are small for their gestational age and with fetal growth restriction (FGR). A premature infant (<37 weeks of gestation) generally has an LBW, which might otherwise have been an appropriate weight if growth had continued normally until fullterm, but could also be a result of FGR. FGR is not an indication for a caesarean section, but FGR infants are likely to suffer from hypoxia and are not responsive to contractions. Thus, it is necessary to select the appropriate time for caesarean section as required, in FGR infants and identify potential risk factors to facilitate early intervention. Antepartum electronic fetal heart monitoring could be used closely during labor, and the delivery mode should be determined according to amniotic fluid volume and the duration of labor.

The prevalence of macrosomia was relatively muted, varying from 4.0% to 2.5%, and the downward trend is consistent with previous reports in China^24^, but the incidence was lower than in the northern part of China^5^. It has been reported that, since 2000, the incidence of macrosomia has been increasing across the United States and in other developed countries^25,26^. From the results of this study, although the proportion of pregnant women with an increased nutritional intake ratio has increased along with the improvement in the economy, the incidence of macrosomia showed a downward trend. This steady downward decline occurred later in Foshan, which may be explained through increased medical and health awareness and improved nutrition, education supervision and advice for all pregnant women, encouraging them to control their weight to within an acceptable range. Foshan is an economically developed city with low infant mortality rates, and the debate about whether caesarean section is needed for macrosomia has focused mainly on avoiding serious consequences such as shoulder dystocia, clavicular fractures, possible postpartum hemorrhage, and brachial nerve injuries. Obstructed labor has resulted in notable adverse outcomes for mothers and infants in developing countries^27^, and cephalopelvic disproportion due to macrosomia is one of the most important outcomes^28^. However, aprenatal diagnosis of suspected macrosomia is challenging. Therefore, it remains controversial whether to perform a caesarean section in cases of suspected macrosomia. For a pregnant woman with an underlying disease, or with a small pelvis or who is malnourished, a caesarean section should be performed to prevent serious consequences, as fetal macrosomia is a major risk factor for infant morbidity and mortality.

Our findings showed that HDCP were strong risk factors associated with LBW, which is consistent with several other studies^29^. This should alert gynecologists to be attentive to an early diagnosis of preeclampsia and eclampsia, which involve the two serious phases of HDCP, through testing mean arterial pressure, undertaking the roll-over test or hematological examinations. Although no consensus on macrosomia standards have been reached, there is evidence that the risk of adverse neonatal outcomes increases when the birth weight is 4000 g or greater^30^. As such, in this study of Asian women, a cut-off point of 4000 g seemed appropriate. In our study, being pregnant with diabetes, older gestational age and maternal age, high parity, and obesity were risk factors strongly associated with macrosomia. With an ageing population and an increasing maternal age following the introduction of the universal two-child policy in China, in addition to growing numbers of people with diabetes and obesity, macrosomia will continue to be an important public health issue in the future.

Electronic health records (EHRs) are an increasingly utilized resource for clinical and public health research, but there are several potential limitations with this study. First, despite this hospital being the largest maternity and child health care hospital in Foshan, with a stable number of more than 13000 deliveries per year, Berkson’s bias is likely, due to this being a single-center study. Therefore, the results might not be generalizable to other cities or countries discussed in this study. Additionally, the hospital’s electronic information system has been upgraded several times between 2005 and 2017, and may have an approximate 5% error rate, with some values missing from previous information. In the data cleaning and analysis phase, we corrected some of the obvious errors and either completed or deleted the missing values, which reduced errors to a minimum. Furthermore, our electronic information system does not provide information about gestational weight gain, which has been associated with macrosomia in previous studies^19^, and this may have resulted in biased estimates. Finally, this paper takes LBW as the research object, rather than the concepts of “small for gestational age” and “intrauterine growth restriction” which were commonly used by obstetrician. All these potential limitations should be considered when interpreting the results of this study.

## Conclusion

Constant urbanization and increasing growth in the national economy, coupled with the lifting of the one-child policy in China, means that the risk factors for LBW and macrosomia, such as hypertension, diabetes, obesity, and an older maternal age of childbearing women, will continue to increase rapidly. Thus, despite the relevant figures having declined slightly in our study, LBW and macrosomia will continue to be a major public health issue. More research aimed at controlling and preventing LBW and macrosomia risk factors and the detrimental outcomes in mothers and perinatal children is needed in China.

## Methods

### Study Population

The city of Foshan, located in South China, is the one of the largest city with a population of nearly 7.4 million of China. The entire study population consisted of 102,526 individuals at least 20 weeks gestation from Southern Medical University Affiliated Maternal & Child Health Hospital of Foshan city, Guangdong province, China between January 2005 and March 2017. All mothers who had singleton births at the hospitals during this period were put into analysis. There were totally 109,780 live births in our study sites. The study excluded 4.708 births with missing birth weight, or newborns outside the range of 20–44 weeks, 2,500 cases of multiple pregnancies, and 46 cases with incredible weight range. Finally, a total of 102,526 cases were included in the study. This hospital was a tertiary university-affiliated medical center with a stable number of approximately 10,000 to 13,000 deliveries per year, which is accounting for 1/8 of total number of newborns in Foshan city. An informed consent was obtained from all participants. In order to ensure patient privacy, our data did not include the patient’s name, phone number, home address, or other sensitive information.

The study was approved by the Human Subjects Committee of the Southern Medical University Affiliated Maternal & Child Health Hospital of Foshan and all methods were performed in accordance with the relevant guidelines and regulations^30^.

### Definition of Variables and Data Extraction

Refered to the standard of the WHO, low birth weight (LBW) is defined as birth weight less than 2500 grams, and subdivided further into very low birth weight (VLBW, <1500 g) and moderately low birth weight (MLBW, 1500 to 2499 g). Macrosomia is defined as birth weight more than 4000 grams. The major covariates studied included the following characteristics of the mother: infant sex [male, female], age [less than 20, 20 to 24, 25 to 30, 30 or more years], mode of delivery [vaginal birth, cesarean section], parity [one, two, three or more], gestational age [<37weeks, ≥37weeks], hypertensive disorder complicating pregnancy(HDCP) or not, diabetes mellitus or not, body mass index (BMI) [<25, 25 to 30, ≥30]. We calculated gestational age on the basis of the actual delivery date in the medical record. HDCP (systolic blood pressure ≥ 140 mmHg and/or diastolic blood pressure ≥ 90 mmHg, with or without proteinuria) can be classified into five groups: (1)gestational hypertension, (2)preeclampsia, (3)eclampsia, (4)superimposed preeclampsia on chronic hypertension and (5) chronic hypertension in pregnancy^31,32^. Diabetes including diabetes mellitus (DM) and gestational diabetes mellitus (GDM). Diagnostic criteria with reference to the recommendation from the Chinese Medical Association Obstetrics Group^33^. GDM was diagnosed when one of the following condition was met: (1) fasting plasma glucose ≥5.8 mmol /L twice or more times; (2) two or more test results are equal or above the following values after a 100 g-load OGTT: fasting, 5.8 mmol/L; 1-hour, 10.6 mmol/L; 2-hour, 9.2 mmol/L; 3-hour, 8.6 mmol/L; (3) 50 g glucose challenge test ≥11.1 mmol/L and fasting plasma glucose ≥5.8 mmol/L. All diagnosis of HDCP and diabetes was confirmed by obstetricians or specialist physician. Body mass index (BMI) was calculated from height and weight before delivery (BMI = weight in kg/height^2^ in meter). To ensure the reliability of data, systematic training was provided for data entry operator for understanding of the study objective and methods.

### Statistical analysis

For descriptive analysis, mean and SD were calculated for continuous variables, and percentages for categorical variables. Birth weight was analyzed by categorizing into four outcome variables: VLBW (<1500 g), MLBW (1500 to 2499 g), normal birth weight (2500 to 4000 g) and macrosomia (≥4000 g). Trend analysis were quantified using the Join point Regression Program (National Cancer Institute), version 4.4.0.0, which uses permutation analysis to fit a series of straight lines on a logarithmic scale to observed rates to estimate annual percent change (APC), resulting in trends of variable length. The Annual Percent Change (APC) is significantly different from zero at the alpha = 0.05 level. Binary logistic regression was used to estimate the relative risk (RR) and corresponding 95% confidence interval (CI) of the association between low birth weight and macrosomia. We mutually adjusted the analysis of the risk factors for LBW and macrosomia for infant sex, maternal age, parity, mode of delivery, gestational age, HDCP, diabetes, body mass index (BMI) in the model. The variables were considered based on literature review for their relevance to LBW and macrosomia and availability of data. *p*-Values of two-sided tests, 0.05 were considered statistically. All analyses were performed with R for Windows version 3.4.1.
